# Towards the identification of a gene for prostrate tillers in barley (*Hordeum vulgare* L.)

**DOI:** 10.1371/journal.pone.0192263

**Published:** 2018-02-08

**Authors:** Yi Zhou, Gaofeng Zhou, Sue Broughton, Sharon Westcott, Xiaoqi Zhang, Yanhao Xu, Le Xu, Chengdao Li, Wenying Zhang

**Affiliations:** 1 Hubei Collaborative Innovation Center for Grain Industry/ School of Agriculture, Yangtze University, Jingzhou, China; 2 Western Barley Genetics Alliance/WA State Agricultural Biotechnology Centre, Murdoch University, Murdoch, Australia; 3 Department of Primary Industry and Regional Development, Government of Western Australia, South Perth, Australia; Institute of Genetics and Developmental Biology Chinese Academy of Sciences, CHINA

## Abstract

Tiller angle, an important agronomic trait, contributes to crop production and plays a vital role in breeding for plant architecture. A barley line V-V-HD, which has prostrate tillers during vegetative growth and erect tillers after booting, is considered the ideal type for repressing weed growth and increasing leaf area during early growth. Genetic analysis identified that the prostrate trait in V-V-HD is controlled by a single gene. A double haploid population with 208 lines from V-V-HD × Buloke was used to map the prostrate growth gene. Ninety-six SNP markers were used for primary mapping, and subsequently, SSR and InDel markers were used for fine mapping. The gene was fine-mapped to a 3.53 Mb region on chromosome 3HL between the markers InDelz3028 and InDelz3032 with 52 candidate genes located in this region. Gene annotation analysis of the 52 genes within the target region indicated that a gene involved in zinc-ion binding (gene ID HORVU3Hr1G090910) is likely to be the candidate gene for prostrate growth in V-V-HD, and is linked to the *denso/sdw* gene. Association analysis showed that prostrate plants were shorter, flowered later.

## Introduction

Barley (*Hordeum vulgare* L.) (2n = 14) is among the world’s earliest domesticated cereal crops [1]. It is widely adapted to diverse environmental conditions and is more tolerant to abiotic stresses than its close relative wheat [2]. Barley is an excellent source of molybdenum, manganese, dietary fiber and selenium [3] and is used for human food, livestock feed and as a raw material in alcohol production [4]. With the increasing global population, the demand for food is expected to double by 2050 [5]. The ideotype breeding might be an effective strategy to further improve crop yield in the future [6,7].

Tiller angle is an important trait for ideal plant architecture as it can significantly affect grain yield [8–10]. Such genotypes occupy more space to increase photosynthetic efficiency, and the dwarf plants are more tolerant trampling than the taller ones [10]. The dynamic tiller angle during a plant’s life cycle can improve the efficiency of water utilization. At the tillering stage, the prostrate growth trait enables plants to occupy a large space for photosynthetic accumulation and weed growth inhibition [11]. After the jointing stage, the tiller angle decrease, plant density increases.

Tiller development is determined by genetic and environmental factors, results in highly phenotypic plasticity for plants to accommodate different environmental conditions [12]. Thus far, several genes associated with barley tiller number, including *cul2* [13], *Int1* [14], *als1* [15], *Int-c* [16], have been identified. However, research on tiller angle in barley is limited. The *denso* gene is well known as one of the most important gene underlying semi-dwarfing characteristics, which has been widely used in European barley breeding programs. Recessive alleles at the *denso* locus showed an association of prostrate early growth habit [17]. However, whether the prostrate growth habit is commonly controlled by the *denso* gene in barley varieties has not been illustrated up to now.

In maize [18–20] and tomato [21], tiller angle is controlled by a lazy gene. The rice tiller angle gene *LA1* is a grass-specific gene that plays a negative role in polar auxin transport (PAT) [22]. Loss of function of *LA1* leads to reduced gravitropism and tiller spread [22,23]. In wild rice, prostrate gene *PROG1* controls tiller angle and tiller number, encoding zinc-finger nuclear transcription factor [24]. *TAC1* is a major QTL on chromosome 9 for tiller angle in rice. Reduced expression of *tac1* promotes asymmetrical growth at the base of the culm [25]. Recently, the *TAC3* and *qTAC8* loci controlling tiller angle have been explored [8,9]. Tiller angle in plants is not only regulated by genetic factors but also by environmental factors [26].

The barley line V-V-HD has a prostrate growth habit during the vegetative phase and erect growth habit during the booting phase. This research aimed to identify candidate genes that control dynamic tiller angle in V-V-HD and analyze the relationship between prostrate growth habit and agronomic traits including tiller number, grains per spike, plant height and flowering time.

## Materials and methods

### Plant materials

**Fig 1:**
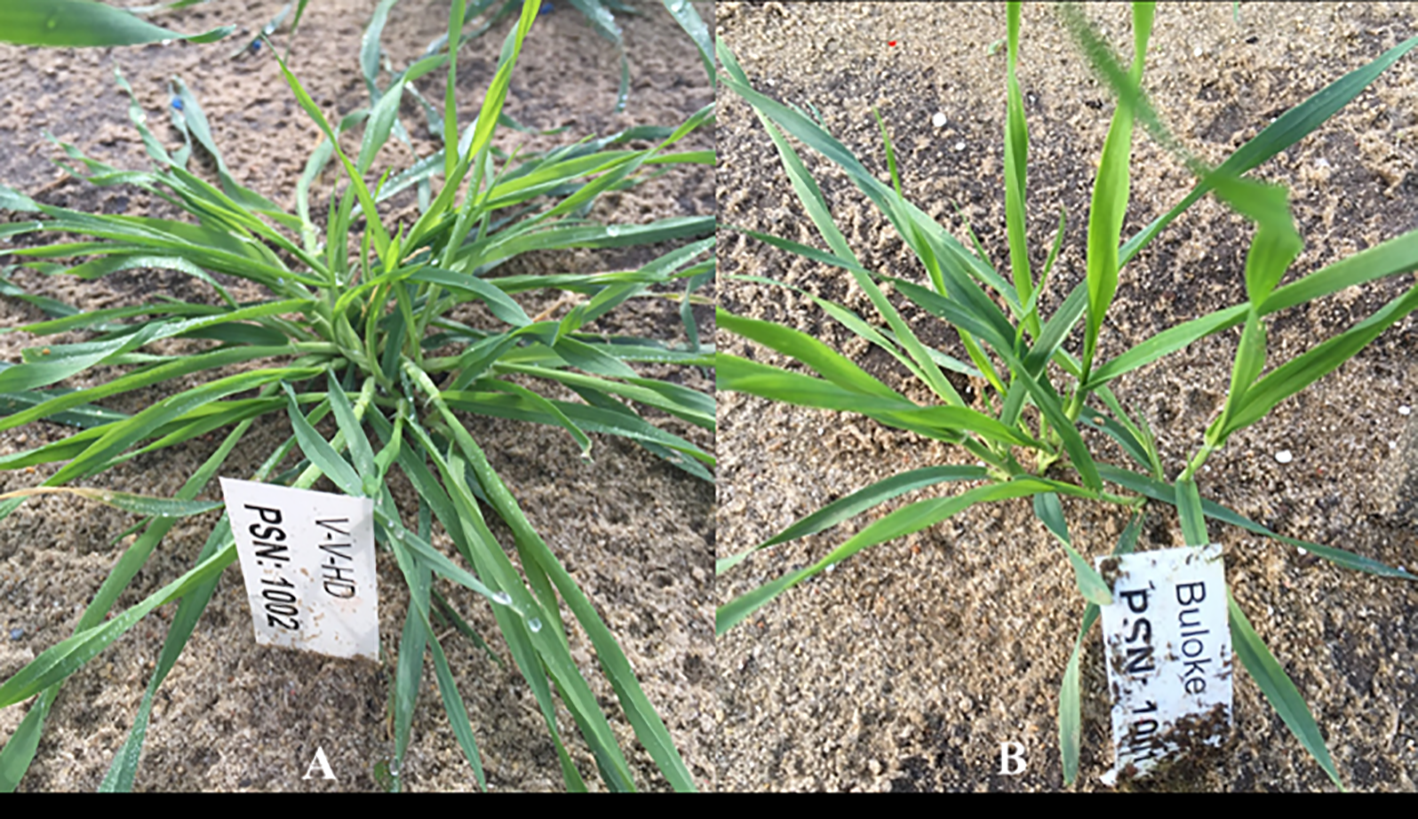
Growth habit of parental lines. Left, prostrate parental line V-V-HD; right, erect growth parental line Buloke.

The barley line V-V-HD was initially selected from a M_2_ population of a malting barley variety Vlamingh (erect growth) treated by 200 gray gamma-radiation. It has a prostrate growth habit during vegetative growth and erect tillers after booting, while Buloke, a major commercial barley variety in Australia, has an erect growth habit (Fig 1). A total of 208 double haploid (DH) lines were developed by anther culture from the F_1_ of V-V-HD × Buloke.

### Phenotype data collection

The DH population was sown in the field at the Department of Agriculture and Food Western Australia (31°59′30″S 115°53′12″E, Kensington, WA) on 19 June 2012 and June 8^th^ 2016. In the 2012 trial, growth habit (erect or prostrate) was recorded three times between August and September. In the 2016 trial, growth habit was recorded on four occasions (19 July, 27 July, 1 August, 11 August). For scoring of prostrate habit, ‘1’ presented as prostrate growth habit, ‘2 presented as erect growth habit, ‘0’ presented as the medium types. Plant height, flowering time, tiller number and grains per spike were measured in the 2016 field trial. Correlation coefficients (*r*) between prostrate growth habit and the measured agronomic traits were calculated.

The DH population and parents were planted in 1 × 5 m plots in a randomized complete block design. Parental and local barley varieties were used as grid controls for spatial analysis. The yield was adjusted according to the Best Linear Unbiased Prediction (BLUP) [27].

### Genotyping and statistical analysis

For primary mapping, 96 single-nucleotide polymorphisms markers (SNPs), distributed on seven chromosomes, were used to genotype 47 DH lines. Of these DH lines, nearly half had a prostrate growth habit. SNP genotyping was performed using the Fluidigm SNP genotyping system. By calculating the correlation determinant between genotypes and phenotypes, the closest marker related to the prostrate trait was identified. To genotype the DH lines, SSR (simple sequence repeat) markers and insertion–deletion markers (InDels) were used to fine map the gene. InDel markers were developed by aligning barley genomic sequences from Morex, Barke, Bowman, and Buloke. InDel markers were designed using the software Geneious 9.0.1.

The PCR reaction contained 1 μl of 10× reaction buffer, 0.3 μl of 50 mM MgCl_2_, 0.2 μl of 10 mM dNTP, 0.2 μl of 10 μM primer, 1 unit Taq polymerase (BIOTAQ DNA polymerase) and 1 μl of 100 ng/μl DNA template. The PCR program was: 95°C for 4 minutes, (94°C for 30 s, 56°C for 30 s and 72°C for 30 s) × 34 cycles, 72°C for 5 minutes. PCR products were separated on 2% agarose gel or 6% polyacrylamide gel electrophoresis (PAGE).

### Genetic map construction

For linkage map construction, the genetic distances between markers were calculated using JoinMap 3.0 [28]. DH1 was selected for population type in the software, and genotypes ‘A’ or ‘B’ were imported into the dataset. Markers were distributed into different linkage groups with Logarithm of odds (LOD) thresholds (from LOD 3 to LOD 10). The final map was validated according to the barley reference genome sequence [29].

## Results

### Segregation of prostrate trait

The prostrate trait (Fig 1) was scored from 208 DH lines of V-V-HD × Buloke population, of which 77 lines had prostrate growth habit, 112 lines had erect growth traits, and 19 lines could not be distinguished. Chi-square test (*p* = 0.0108) demonstrated that the ratio of prostrate: erect growth type fit the 1: 1 segregation ratio in the DH population. The result confirmed that the prostrate trait in V-V-HD was controlled by a single locus.

### Correlation analysis between prostrate and agronomic traits

**Fig 2:**
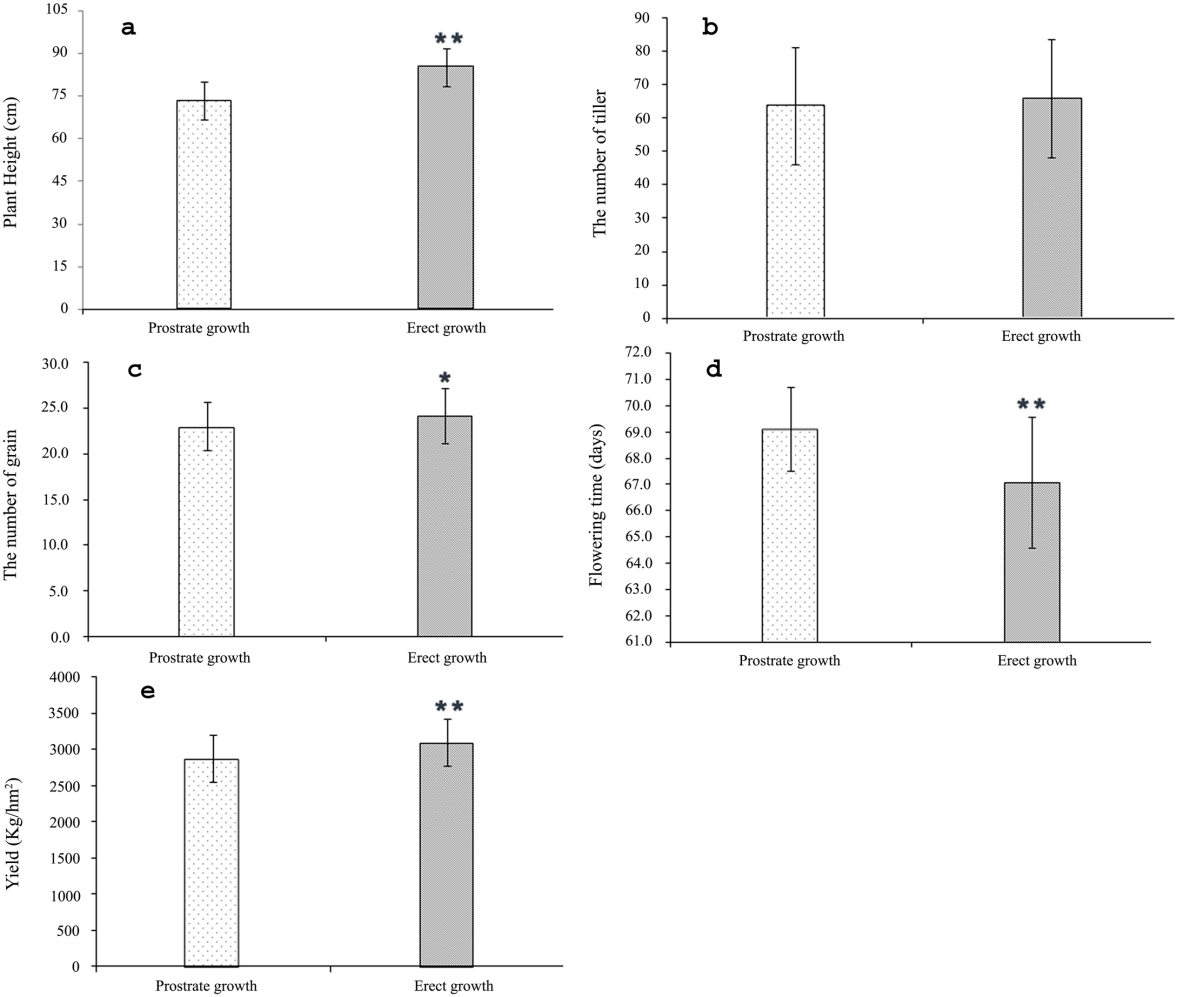
Measured agronomic traits in prostrate and erect barley plants. (a) plant height, (b) tiller number, (c) grains per spike, (d) flowering time, and (e) yield (* p < 0.05, ** p < 0.01).

Mean plant height was 73.2 cm and 84.9 cm for prostrate and erect plants, respectively (Table 1, Fig 2). The correlation coefficient for prostrate growth habit and plant height was significant with *r* = -0.66 (*p* < 0.01) (Table 1). The prostrate growth habit had no significant relationship on tiller number with *r* = -0.06 (*p* > 0.05), with average tiller numbers of 63.6 and 65.8 per row in prostrate and erect plants, respectively (Table 1, Fig 2). The prostrate growth habit affected grain number per spike with *r* = -0.20 (*p* < 0.05) (Table 1), with 22.9 and 24.1 grains per spike in prostrate and erect plants, respectively (Fig 2). The average yields of prostrate and erect barley were 2867.9 kg/hm^2^ and 3093.8 kg/hm^2^, respectively. The correlation coefficients between the prostrate trait and flowering time and yield were 0.42 (*p* < 0.01) and -0.32 (*p* < 0.01), respectively (Table 1). Prostrate plants flowered later (*p* < 0.01) and had lower grain yields (*p* < 0.01) than erect plants (Table 1, Fig 2). Taller plants had more grains per spike and higher yields than shorter plants, with *r*-values of 0.37 (*p* < 0.01) and 0.34 (*p* < 0.01), respectively. The number of tillers also had a positive effect on grain yield (*r* = 0.28).

**Table 1:** Correlation coefficients between prostrate growth habit and agronomic traits in barley.

**Trait**	**Prostrate habit**	**Plant height**	**Tiller number**	**Grains per spike**	**Flowering time**	**Yield**
**Prostrate habit**	1					
**Plant height**	–0.66**	1				
**Tiller number**	–0.06	0.17	1			
**Grains per spike**	–0.20*	0.37**	0.14	1		
**Flowering time**	0.42**	–0.25**	–0.02	–0.13**	1	
**Yield**	–0.32**	0.34**	0.28**	0.04	–0.19*	1

* *p* < 0.05,

** *p* < 0.01

### Preliminary mapping of the prostrate gene

**Fig 3:**
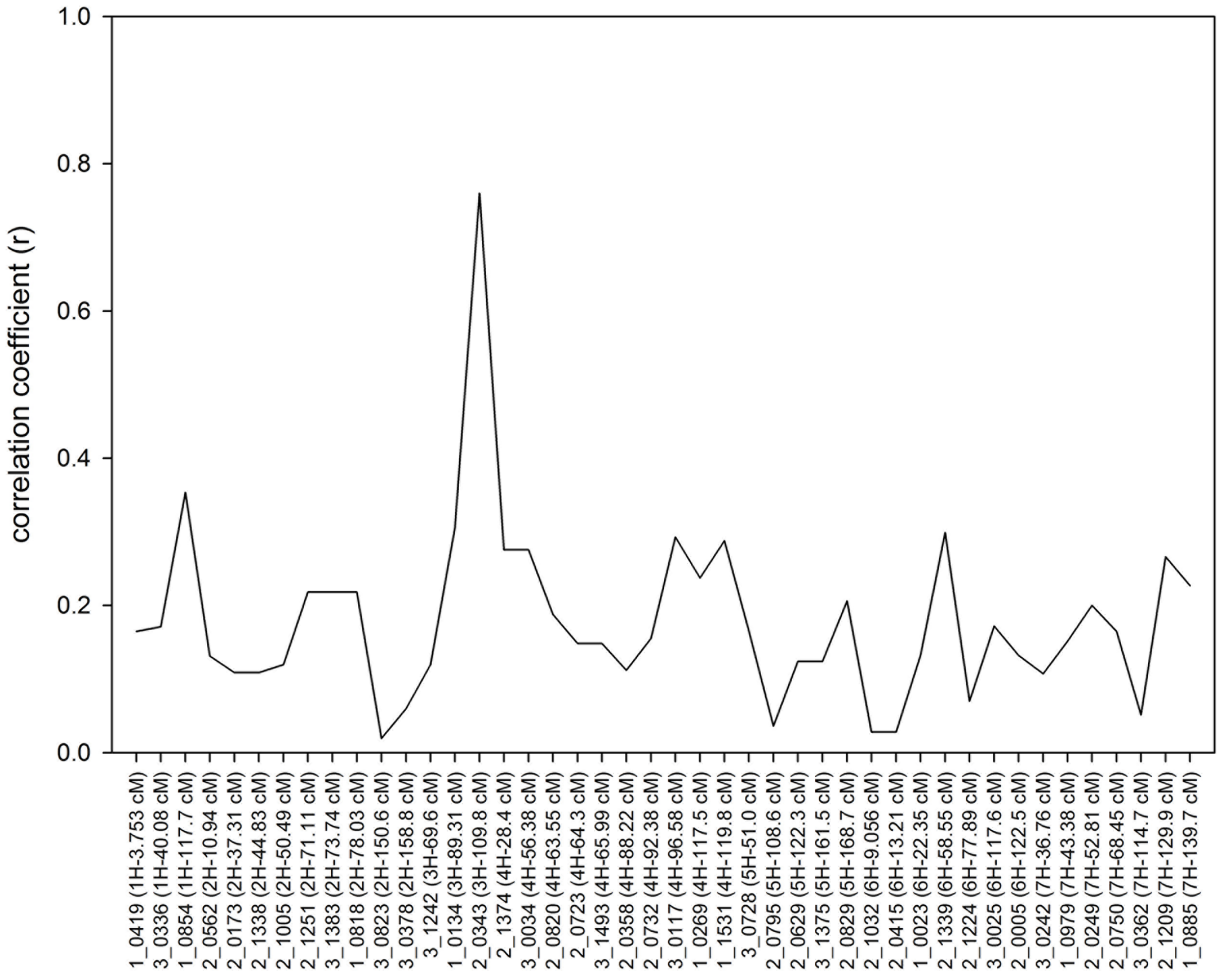
Correlation coefficients (*r*) between prostrate growth and 44 SNP markers. Forty-two DH lines were used in this analysis.

Forty-four of 96 SNP markers [30] showed polymorphism between V-V-HD and Buloke. Forty-seven lines were initially used to map the prostrate trait gene. Of these, 23 lines had erect growth, and 24 lines had prostrate growth. The correlation coefficients between phenotype and genotypes were calculated. The marker 2_0343 (109.84 cM) on chromosome 3H had the highest correlation coefficient of 0.76 and was thus identified as the closest marker to the gene (Fig 3). For marker 2_0343, 37 of the 42 lines with genotypes had consistent phenotypes. The preliminary result indicates that the prostrate gene is located on chromosome 3H.

### Fine mapping of the prostrate gene

**Fig 4:**
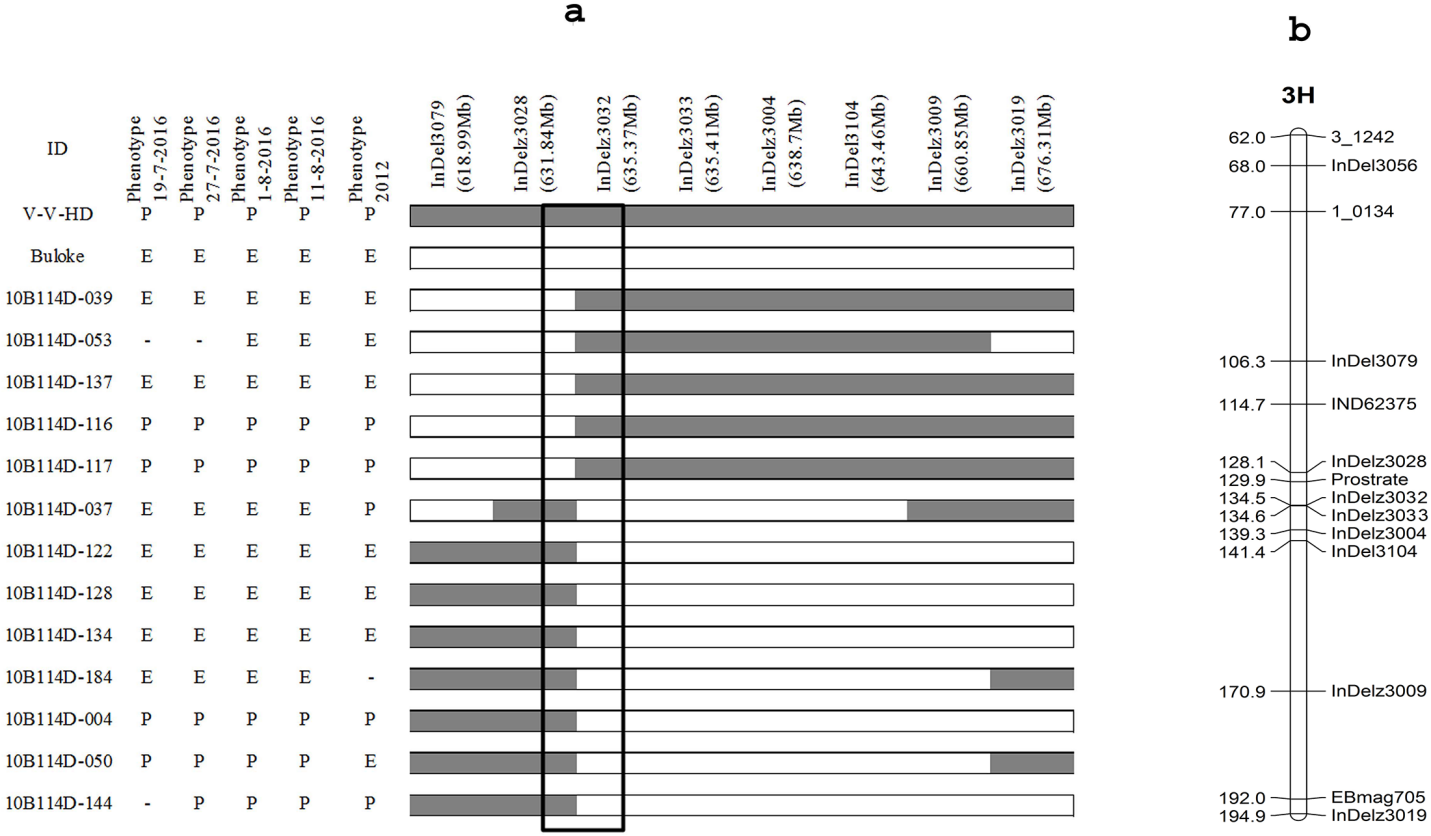
Genotypes and phenotypes of the recombinant lines of the V-V-HD × Buloke DH population and chromosome 3H linkage map. (a) Recombinant lines used for fine mapping, (b) Linkage map of chromosome 3H. E is erect growth; P is prostrate growth; “-” is unclear phenotype.

All 208 DH lines from the V-V-HD × Buloke population were used to fine map the prostrate gene. One hundred InDel markers were designed in the region around marker 2_0343, based on sequence alignments of Morex, Bowan, Barke and Buloke [29]. The primer sequence, amplicon size, and physical map location are summarized in S1 Table. Polymorphic markers were used to genotype the DH population. The prostrate gene was mapped between two markers InDelz3028 and InDelz3032 (Fig 3a). The genetic map locations of the markers were consistent with the physical map (Fig 4a and 4b) [29]. Thirteen recombinant individuals were located between these two markers. The lines 10B114D-039, 10B114D-053, and 10B114D-137 had erect growth habit but prostrate genotype in marker InDelz3032; and lines 10B114D-004, 10B114D-050, and 10B114D-144 had the prostrate growth trait but erect genotype in marker InDelz3032; thus, the correct boundary for the target region was InDelz3032. The lines 10B114D-037 10B114D-122, 10B114D-128, 10B114D-134, and 10B114D-184 had the erect growth trait, but a V-V-HD genotype for marker InDelz3028, and lines 10B114D-116 and 10B114D-117 had a prostrate growth habit but Buloke genotype for marker InDelz3028; thus, the left border was InDelz3028 (Fig 4a). By analyzing the recombinant lines, the target gene was mapped on chromosome 3HL between the markers InDelz3028 (631.84 Mb) and InDelz3032 (635.37 Mb) (Fig 4a).

### Gene annotation

Information on the candidate genes in the target genetic region was extracted from the new barley genome annotation [29], which had 52 genes between markers InDelz3028 (631.84 Mb) and InDelz3032 (635.37 Mb). We then obtained the GO (gene ontology) [31] information on the barley sequence. Using WEGO [32], the 52 genes were further divided into nine clusters, including biosynthetic process, cell wall-related, metabolic process, protein modification, transcription, translation, transport, unknown and others (S2 Table). Of these genes, one gene encodes a gibberellin 20-oxidase which is the functional gene of the *denso*/*sdw* with dwarf and prostrate growth habit. The second gene is a transcription factor and encodes a zinc-ion binding protein, which is homologous to the rice prostrate gene *PROG1* [24]. These two genes were identified as the candidate genes to control the prostrate growth habit in V-V-HD.

## Discussions and conclusions

### Relationship between prostrate growth and agronomic traits

Didon [11] and Tan et al. [10] indicated that the prostrate growth habit could conserve water repress weed growth, occupy more space at seedling stage, and thus have higher photosynthetic efficiency, stronger trampling tolerance, which generally results in high yield. The present study showed that barley with a prostrate growth habit had lower grain yields and flowered later than those with an erect growth habit (Fig 2e). Furthermore, the prostrate growth trait reduced grain number per spike and plant height but had no significant effect on tiller number. In rice, the grass-specific genes *LA1*, *TAC1* and *TAC3* controlled tiller angle but not tiller number [9,22,25]. On the other hand, the gene *PROG1* in rice controlled both tiller angle and tiller number [24]. However, it is clear that the results of this study did not meet our expectations for V-V-HD as the ideal plant type with higher yield potential. Several reasons may explain these results: (i) Buloke is a commercial high-yielding variety and genetic drag linked to the prostrate gene limited yield potential; (ii) the trial site was in a Mediterranean environment with limited rainfall after booting stage, which resulted in higher ratio of tiller abortion and less kernels per spike for the prostrate type due to late flowering; (iii) seeding density and weed management affect prostrate trait expression and (iv) low temperature at seedling stage may delays its transfer from vegetation to reproduction growth for the prostrate type which results in not enough time for booting of stems and development of spikes and finally the later flower, shorter plant, less kernels per spike and low yield. Further research is required to address these possibilities. The molecular markers and candidate genes identified in this study will provide useful tools for answering these questions.

### Environment-dependent phenotyping of prostrate growth

The prostrate growth habit is controlled by both genetic and environmental factors. V-V-HD did not have a prostrate growth habit when sown in summer in a glasshouse (data not shown), but this was evident when sown in winter. Unexpectedly, two lines, 10B114-137 and 10114B-050, showed different growth habits in 2012 and 2016, and 19 lines showed intermedium phenotype. Environmental factors, such as temperature and sunlight, may have had a significant effect on phenotyping expression or additional genes may modify the prostrate trait expression, which need further investigation.

The *denso*/dwarfing gene in a previous study showed a distinctive prostrate juvenile growth habit in barley [17], which is located at 634.08 Mb on chromosome 3H based on the barley reference genome sequence [29] and the function of the *denso* gene [33]. In the present study, mapping results indicated a close link between the *denso* gene and the prostrate gene. Kuczynska et al. [34] found that the barley varieties with the *sdw1/denso* gene showed prostrate growth habit. This raises the question whether the *denso/sdw* gene control prostrate growth in V-V-HD. Allele-specific markers for the *denso* locus have been developed recently [35]. Our test excluded that V-V-HD contains one allele of the *denso/sdw* gene. In rice, prostrate growth was controlled by a zinc binding site gene *PROG1* [10,24,27]. Within the fine mapping region, it is interesting to note that the presence of a gene named AK360532 on barley chromosome 3HL (633.53 Mb), which also encodes the zinc binding site. This gene might be the candidate gene for prostrate growth habit, but further research is needed to characterize this gene.

This is the first report to fine map the prostrate growth trait gene in barley. This gene was mapped on chromosome 3HL near marker InDelz3032, and fine-mapped to a 3.53 Mb region. The prostrate growth habit in barley produced shorter plants that flowered later, had fewer grains per spike and lower grain yields than erect barley plants.

## Supporting information

S1 Table Summary of InDel primers and their distribution in different genomic regions. (XLSX)

S2 Table The detailed gene list of WEGO annotations of 52 candidate genes. (XLSX)
